# Investigation of Effectiveness of Pulsed Radiofrequency With Multifunctional Epidural Electrode for Low Back Pain

**DOI:** 10.7759/cureus.20239

**Published:** 2021-12-07

**Authors:** Leman Gokcenur Gulduren Aydın, Selcan Akesen, Yunus Gurkan Turker, Alp Gurbet, Vildan Kılıç Yılmaz

**Affiliations:** 1 Anesthesiology and Reanimation, Kastamonu Training and Research Hospital, Kastamonu, TUR; 2 Anesthesiology and Reanimation, Bursa Uludag University Faculty of Medicine, Bursa, TUR; 3 Pain Management, Bursa Uludag University Faculty of Medicine, Bursa, TUR; 4 Pain Management, Kocaeli Pain Management Center, Kocaeli, TUR

**Keywords:** pain on vas, multifunctional epidural electrode, pulsed radiofrequency, spinal stenosis, lumbar disc hernia

## Abstract

Aim: Low back pain affects many people at some point in their life. Whenever pharmacologic and other conservative treatments of chronic pain fail, ablative and interventional methods are attempted on the assumption that interrupting nerve conduction prevents central pain cognition. Pulsed radiofrequency using multifunctional epidural electrodes can be used for multiple etiologies of chronic low back and leg pain with a low complication rate and minimal side effects. Methods: The records of the 188 patients who underwent pulsed radiofrequency with multifunctional epidural electrode between October 2014 and March 2017 in Algology clinic were examined retrospectively. Visual analogue scale (VAS) for pain, response to straight leg raising test (SLR), lumbar range of motion, analgesic use, patient satisfaction score, need for open operation or other interventional procedure were collected.

Results: VAS and SLR tests were found to be significantly improved compared with the preoperative values. The VAS scores at the 10th day and first, third and sixth months were significantly decreased compared to baseline scores (p<0.001). Also, SLR tests were significantly improved compared to baseline scores at the same intervals (p<0.001).

Conclusion: Pulsed radiofrequency with multifunctional epidural electrode is a safe and effective method for low back pain which is caused by several pathologies.

## Introduction

Low back pain is a condition that affects many people in a certain period of their lives. Most of the patients (> 85%) applying to primary care due to low back pain have non-specific low back pain, in other words, the underlying cause of their low back pain cannot be defined precisely [1,2].

Low back pain has the highest prevalence in people aged from 40 to 80 years and it has been observed that the prevalence of chronic low back pain has changed over the years due to the change in lifestyles and technology. Two studies have shown that the prevalence of chronic low back pain has doubled over time [3,4]. This may be a reflection of the essential changes in the business world and lifestyles. Extensive use of computers and other technologies in the workplace and at home has increased sedentariness, which is a risk factor for acute and chronic low back pain due to muscle weakness [5].

The cause of low back pain is mostly non-specific [6]. The leading causes may be grouped under the titles of mechanical and rheumatological pathologies [6]. It should be determined in the differential diagnosis whether pain is mechanical or inflammatory [6]. The most common mechanical causes include: osteoarthritis of the lumbar vertebrae, disc herniation, spondylolisthesis, lumbar spinal stenosis, and diffuse idiopathic skeletal hyperostosis [6]. In 1-5% of cases, patients have an underlying condition such as cancer, osteoporosis, fracture, systemic inflammatory disease or other serious problems (“red flags”) [7].

Primary care for the treatment of low back pain includes non-invasive treatment and non-drug methods, such as patient education and suggestions on active life and exercise [7]. Nevertheless, patients that have low back pain accompanied by severe and progressive loss of motor strength or signs of cauda equine should be immediately referred to surgery [8].

Applying epidural or transforaminal corticosteroid injections and dorsal root ganglion (DRG) radiofrequency ablation are invasive treatment options in cases where conservative treatments are insufficient [9]. The flexible multifunctional electrode (the PASHA electrode), which was developed in 2003, is based on the rationale that the dorsal horn plays a central role in modulating nociceptive inputs to the central nervous system [10].

## Materials and methods

Following the Ethics Committee of Uludag University Faculty of Medicine approval (2017-17/45), the study was performed as the retrospective analysis of the medical files of patients admitted to the Clinics of the Algology Department and underwent pulsed radiofrequency (PRF) with multifunctional epidural electrode between October 2014 and March 2017. Patients who had low back pain with unilateral/bilateral radicular pain due to various aetiologies, who had no improvement of pain despite medical treatment and other standard treatment methods, and who were regarded as Class I-III according to the American Society of Anaesthesiologists (ASA) classification were included in the study. We investigated post-procedure Visual Analogue Scale (VAS) scores, response to straight leg raise (SLR) test, range of motion (ROM) of the lumbar joints, use of analgesics, and patient satisfaction level, and whether the patient had any operation or invasive procedure after PRF with multifunctional epidural electrode.

Data of 188 patients meeting the inclusion criteria were examined. Thirty-six patients who did not show up for the post-procedure follow-up visit or whose data could not be accessed were excluded from the study. As a result, the data of 152 patients were evaluated within the scope of the study.

Age, gender, BMI, ASA class, comorbidities and pain duration were recorded for each patient. If they had a history of surgery or neuropathic complaints were also recorded. The patients’ VAS scores before the procedure and after the procedure and at the post-procedural 10th day and first, third and sixth months, response to straight leg raise test before and after the procedure, analgesic use before and after the procedure, and the range of motion values measured before and after the procedure were recorded. After the procedure, patient satisfaction was recorded on a 0-4 scale (very bad: 0, bad: 1, good: 2, very good: 3, excellent: 4). In our study, the range of motion was measured with a goniometer, and the straight leg raise test was performed as described above and the results were recorded in angles.

Normal distribution of variables was assessed using the Shapiro-Wilk test. Continuous variables are expressed with the median (minimum-maximum) and average±standard deviation values. Categorical variables are expressed as % (n=). The dependent paired samples Wilcoxon test was used for comparisons between the groups generated according to the results of the normal distribution tests. Statistical Package for Social Sciences (SPSS) version 21 (IBM Corp., Armonk, NY, USA) was used for statistical analyses. p<0.05 was considered statistically significant.

Pulsed radiofrequency application with multifunctional epidural electrode (PASHA electrode)

After establishing peripheral vascular access, we apply electrocardiography (ECG), pulse-oximetry (SpO2) and non-invasive blood pressure monitoring for patients in accordance with the recommendations of ASA. Patients are placed in the prone position and a neutral electrode is located on the appropriate site. The intervention site is sterilized with antiseptic solution and covered. Sterile conditions are maintained during the procedure. Sacral hiatus is identified, and 3 ml of 2% prilocaine solution is applied to the subcutaneous and deep tissues as the local anaesthetic. Epidural space is accessed using a 14-gauge introducer cannula. Radiopaque material is injected through the cannula and its location is confirmed with fluoroscopy. The multifunctional electrode is advanced through the cannula and placed at the targeted spinal level, dorsal column of medulla spinals or cauda equina nerves, according to MRI, in the craniolateral direction. Its location is checked with anteroposterior, oblique and lateral fluoroscopic images. The proximal end of the catheter is attached to the radiofrequency generator in a sterile way. After observing proper placement, stimulation is applied at 0.8 V, 50 HZ sensory stim, and the location of the catheter is confirmed when patients feel a stimulation in their symptom area; the point of application is determined. PRF is applied at 42 °C for 240-360 seconds with an active phase of 20 milliseconds and a silent period of 480 milliseconds. After the application, the catheter is withdrawn. Patients are followed up for four to six hours for a loss in motor function, sensation and other complications. On the follow-up visit, patients’ VAS scores, SLR test results, joint range of motion, and satisfaction levels are recorded.

## Results

Demographic data and distribution of ASA scores of 152 patients are shown in Table 1 and Table 2.

**Table 1:** Demographic Data

	Age (year) Median (Min-Max)	Weight (kg) Median (Min-Max)	Height (cm) Median (Min-Max)
Female (n=96)	65 (35-89)	76 (50-105)	159.5 (145-182)
Male (n=56)	58 (28-84)	81 (60-115)	172.0 (156-185)

**Table 2:** Distribution of American Society of Anaesthesiologists (ASA) Scores

ASA I	ASA II	ASA III
39.5% (n=60)	57.2% (n=87)	3.3% (n=5)

Most common diagnoses in patients receiving pulsed radiofrequency with multifunctional epidural electrode were respectively lumbar disc herniation (LDH) (60.52%, n=92), spinal stenosis (30.92%, n=47), and spondylolisthesis plus spinal stenosis (8.55%, n=13).

The median VAS score of 152 patients examined in the study before treatment was 6 (4:10) (average±standard deviation: 6.59±0.99). The VAS scores evaluated on day 10 and months one, three, and six after treatment were significantly lower as compared to the VAS values obtained before treatment (p<0.001, p<0.001, p<0.001, respectively) (Table 3). 

**Table 3:** Pre-treatment (Tpre) and Post-treatment Visual Analogue Scale (VAS) Scores for All Patients

	Median	Min	Max	Mean ±SD	Median	Min	Max	Mean ±SD	P
Tpre – Day 10	6	4	10	6.59 ±0.98	3	0	8	3.28 ±1.79	<0.001
Tpre – Month 1	6	4	10	6.59 ±0.98	3	1	8	3.28 ±1.82	<0.001
Tpre – Month 3	6	4	10	6.59 ±0.98	3	1	8	3.22 ±1.85	<0.001
Tpre – Month 6	6	4	10	6.59 ±0.98	3	1	8	3.32 ±1.85	<0.001

In the study, the Wilcoxon test was used to compare the pre-treatment VAS values with the VAS values measured 10 days and one, three, and six months after treatment (Table 4). The pre-treatment VAS values were compared with the values at the 10th day and the first, third and sixth months after the treatment in patients diagnosed with LDH, and the post-treatment scores were found to be significantly lower (p<0.001, p<0.001, p<0.001, and p<0.001, respectively). When the pre-treatment VAS values were compared with the values at the 10th day and the first, third and sixth months after the treatment in patients with spinal stenosis, the post-treatment scores were significantly lower (p<0.001, p<0.001, p<0.001, and p<0.001, respectively.). When the pre-treatment VAS values were compared with the values on the 10th day and in the first, third and sixth months after treatment in patients with spondylolisthesis plus spinal stenosis, the post-treatment values were found to be significantly lower (p=0.002, p=0.002, p=0.002, p=0.002, respectively).

**Table 4:** Change in the Visual Analogue Scale (VAS) Scores by Diagnoses LDH: lumbar disc herniation

	LDH n=92		Spinal Stenosis n=47		Spondylolisthesis and Spinal Stenosis n=13	
	Median (min-max) Avg.±SD		Median (min-max) Avg.±SD		Median (min-max) Avg.±SD	
Tpre – Day 10	6 (5-10) 6.61±0.99	3 (0-8) 3.36±1.86	6 (4-8) 6.51±0.93	3(1-7) 3.23±1.69	6 (6-10) 6.62±1.19	3 (1-7) 2.85±1.72
P	p<0.001^a^		p<0.001^a^		p=0.002^a^	
Tpre – Month 1	6 (5-10) 6.61±0.99	3 (1-8) 3.35±1.83	6 (4-8) 6.51±0.93	3 (1-8) 3.28±1.85	6 (6-10) 6.62±1.19	2 (1-7) 2.77±1.73
P	p<0.001^a^		p<0.001^a^		p=0.002^a^	
Tpre – Month 3	6 (5-10) 6.61±0.99	3 (1-8) 3.21±1.80	6 (4-8) 6.51±0.93	3 (1-8) 3.28±1.91	6 (6-10) 6.62±1.19	2 (1-7) 3.08±2.10
P	p<0.001^a^		p<0.001^a^		p=0.002^a^	
Tpre – Month 6	6 (5-10) 6.61±0.99	3 (1-8) 3.30±1.82	6 (4-8) 6.51±0.93	3 (1-8) 3.53±1.93	6 (6-10) 6.62±1.19	2 (1-7) 2.69±1.75
P	p<0.001^a^		p<0.001^a^		p=0.002^a^	

We compared the pre-treatment straight leg raise test values with the post-treatment straight leg raise test values according to diagnosis. In the patients with LDH, the post-treatment SLR values were found to be significantly higher than the pre-treatment SLR values (p<0.001). In the patients with spinal stenosis, the post-treatment SLR values were also significantly higher compared to the pre-treatment SLR values (p<0.001). furthermore, the post-treatment SLR values of patients with spondylolisthesis plus spinal stenosis were also found to be significantly higher compared to the pre-treatment SLR values (p=0.083).

We compared the pre-treatment range of motion for joint flexion with the post-treatment range of motion by diagnosis. In the group diagnosed with LDH, the post-treatment flexion ROM was found to be significantly increased compared to pre-treatment (p=0.003). 

In the group of patients with spinal stenosis, no significant difference was found between pre-treatment and post-treatment ROM values in flexion (p=0.102). Similarly, there was no significant difference between pre-treatment and post-treatment ROM values for flexion in patients with spondylolisthesis plus spinal stenosis (p=0.157).

We compared the pre-treatment range of motion for joint extension with the post-treatment range of motion by diagnosis. In the patients with LDH, the post-treatment ROM values for extension were found to be significantly higher as compared to the pre-treatment ROM values (p<0.001). On the other hand, no significant difference was found between pre-treatment and post-treatment extension ROM values in the group diagnosed with spinal stenosis (p=0.066). There was no significant difference between pre-treatment and post-treatment ROM values for flexion in the group with spondylolisthesis plus spinal stenosis (p=0.059).

We made a comparison between the pre-treatment range of motion for lateral flexion and the post-treatment range of motion for lateral flexion according to patients’ diagnoses. In the group diagnosed with LDH, the post-treatment ROM values for lateral flexion were found to be significantly higher than the pre-treatment ROM values (p<0.002). Nevertheless, there was no significant difference between pre-treatment and post-treatment lateral flexion ROM values in the group diagnosed with spinal stenosis (p=0.046). There was no significant difference between pre-treatment and post-treatment ROM values for lateral flexion in the group with spondylolisthesis plus spinal stenosis (p=0.157).

**Table 5:** Straight leg raise (SLR) test (degree), joint range of motion for flexion (ROM, degree), joint range of motion for extension (ROM, degree), joint range of motion for lateral flexion (ROM, degree) by diagnosis. LDH: lumbar disc herniation

	LDH (n=92)		Spinal Stenosis (n=47)		Spondylolisthesis and Spinal Stenosis (n=13)	
	Median (min-max) Avg ±SD		Median (min-max) Avg ±SD		Median (min-max) Avg ±SD	
Tpre SLR–	62,5 (20-80)	70 (30-90)	70(15-80)	70(30-85)	70(45-80)	70(45-80)
Tpost SLR	58,85 ±15,44	66,63±14,18	62,55±14,88	69,04±11,16	68,46±8,26	70,77±8,86
	p<0,001^a^		p<0,001^a^		p=0,083^a^	
Tpre flexion	50(30-60)	55(30-60)	50(30-60)	50(30-60)	50(35-60)	50(35-60)
ROM – Tpost	49,13±10,07	50,05±9,76	47,55±10,67	47,98±10,51	45,38±9,67	46,15±8,93
flexion						
ROM						
	p=0,003^a^		p=0,102^a^		p=0,157^a^	
Tpre	20(10-35)	20(10-35)	20(10-35)	20(10-35)	15(10-35)	20(10-35)
extension	22,33±9,24	23,37±8,77	21,60±9,44	22,34±9,37	16,54±6,57	18,46 ±5,91
ROM – Tpost						
extension						
ROM						
	p<0,001^a^		p=0,066^a^		p=0,059^a^	
Tpre lateral	15(5-20)	15(10-20)	15(5-20)	15(10-20)	15(10-20)	15(10-20)
flexion	14,89±4,44	15,59 ±4,11	14,68±4,47	15,11±4,23	13,08 ±3,25	13,85±2,99
ROM – Tpost						
lateral						
Flexion ROM						
p=0,002^a^		p=0,046^a^		p=0,157^a^	

Whereas 113 patients (74.3%) were using analgesic before treatment, this was decreased to 83 patients (54.6%) after the procedure. It was observed that the number of patients undergoing a physiotherapy protocol was 58 (38.2%) before treatment and it decreased to 11 (7.2%) after treatment. The number of patients who received interventional treatment by algology physicians before the application of pulsed radiofrequency with multifunctional epidural electrode was 65 (42%), and 87 (57%) did not have any history of surgery.

There were 42 patients (27.6%) with a history of surgery before treatment, whereas 24 patients (15.8%) underwent surgery after treatment. When the patients who had history of surgery before the treatment were examined, it was observed that the VAS scores prior to the application of the pulsed radiofrequency with multifunctional epidural electrode were significantly higher than the VAS scores recorded on the 10th day and in the 1st, 3rd and 6 months after the treatment (p<0.001; p<0.001; p<0.001; p<0.001, respectively). Moreover, when the patients who did not have history of surgery before the treatment were examined, the VAS scores prior to the application of the pulsed radiofrequency with multifunctional epidural electrode were also significantly higher than the VAS scores recorded on the 10th day and in the first, third and sixth months after the treatment (p<0.001; p<0.001; p<0.001; p<0.001, respectively) (Table 6).

**Table 6:** Change in the Visual Analogue Scale (VAS) scores of the patient groups with and without a history of surgery before treatment

	Patients with a history of surgery (n=42)		Patients without a history of surgery (n=110)	
	Median (min-max) Avg.±SD		Median (min-max) Avg.±SD	
Tpre – Day 10	7 (5-10) 6.95±1.05	4 (1-7) 4.14±1.77	6 (4-10) 6.45±0.92	3 (0-8) 2.95±1.69
P	p<0.001		p<0.001	
Tpre – Month1	7 (5-10) 6.95±1.05	4 (1-8) 4.07±1.87	6 (4-10) 6.45±0.92	3 (1-8) 2.98±1.17
P	p<0.001		p<0.001	
Tpre – Month3	7 (5-10) 6.95±1.05	4 (1-8) 3.89±1.87	6 (4-10) 6.45±0.92	2 (1-8) 2.97±1.78
P	p<0.001		p<0.001	
Tpre – Month6	7 (5-10) 6.95±1.05	4 (1-8) 3.93±1.90	6 (4-10) 6.45±0.92	3 (1-8) 3.09±1.78
P	p<0.001		p<0.001	

The pre-treatment and post-treatment straight leg raise test scores were compared in the groups with and without a surgical history prior to the application of pulsed radiofrequency with multifunctional epidural electrode. The post-treatment SLR values were found to be significantly higher than the pre-treatment SLR values in the group that had a history of surgery (p<0.001). Similarly, the post-treatment SLR values of the group without a history of surgery were significantly higher than the pre-treatment SLR values (p<0.001).

Furthermore, the pre-treatment range of motion for flexion was compared with the post-treatment range of motion in patients with and without a surgical history prior to the application of pulsed radiofrequency with multifunctional epidural electrode. In the group with a history of surgery, the post-treatment ROM values for flexion were significantly higher compared to the pre-treatment values (p<0.034). In the group without a history of surgery, the post-treatment ROM values for flexion were significantly higher compared to the pre-treatment values (p=0.003).

We also compared the pre-treatment range of motion for extension with the post-treatment range of motion in patients with and without a surgical history before the application of pulsed radiofrequency with multifunctional epidural electrode. There was no significant difference between pre-treatment and post-treatment extension ROM values in the group that had surgery prior to the treatment (p=0.066). On the other hand, the post-treatment ROM values for extension were significantly higher compared to the pre-treatment values in the group without a history of surgery (p<0.001).

A comparison was also made between the pre-treatment and post-treatment range of motion levels for lateral flexion in patients with and without a surgical history prior to the application of pulsed radiofrequency with multifunctional epidural electrode. No significant difference was determined between the pre-treatment and post-treatment ROM values for lateral flexion in the group that had surgery prior to the treatment (p=0.083). In the group without a history of surgery, the post-treatment ROM values for lateral flexion were significantly increased compared to the pre-treatment values (p<0.001) (Table 7).

**Table 7:** Change in the straight leg raise (SLR) test (degree) scores and range of motion (ROM, degree) of the patient groups with and without a history of surgery before treatment

	Patients with a history of surgery (n=42)		Patients without a history of surgery (n=110)	
	Median (min-max) Avg.±SD		Median (min-max) Avg.±SD	
Tpre SLR –	60 (20-80)	70 (30-90)	70 (15-80)	70 (30-85)
Tpost SLR	55.60±15.93	62.98±15.92	62.82±14.19	69.55±11.16
	p<0.001^a^		p<0.001^a^	
Tpre flexion	50 (30-60)	50 (30-60)	50 (30-60)	52.5 (30-60)
ROM – Tpost	47.00±10.71	47.74±10.43	48.82±10.04	49.59±9.77
flexion ROM				
	p=0.034^a^		p=0.003^a^	
Tpre extension	20 (10-35)	20 (10-35)	20 (10-35)	20 (10-35)
ROM – Tpost	20.71±8.80	21.55±8.73	21.95±9.36	23.05±8.85
extension				
ROM				
	p=0.066^a^		p<0.001^a^	
Tpre lateral	15 (10-20)	15 (10-20)	15 (5-20)	15 (10-20)
flexion ROM	14.76 ±4.12	15.12±4.05	14.64±4.47	15.36±4.10
– Tpost lateral				
flexion ROM				
	p=0.083^a^		p<0.001^a^	

The number of patients that had neuropathic pain complaints before treatment was 58 (38.2%), and the number of patients whose pain had no neuropathic feature was 94 (61.8%). When the patients who had neuropathic pain were analysed, it was observed that their VAS scores prior to the application of the pulsed radiofrequency with multifunctional epidural electrode were significantly higher than the VAS scores measured 10 days and one, three and six months after the treatment (p<0.001; p<0.001; p<0.001; p<0.001, respectively). Moreover, patients who had low back pain without radicular leg pain were examined; the VAS scores prior to the application of the pulsed radiofrequency with multifunctional epidural electrode were significantly higher compared to the VAS scores on the 10th day and in the first, third and sixth months after the treatment (p<0.001; p<0.001; p<0.001; p<0.001, respectively).

There were no complications in the 152 patients whose data we examined. None of the 152 patients developed neurological dysfunction after the procedure.

Patient satisfaction score was 1 in 21 (13.8%), 2 in 26 (17.1%), 3 in 39 (25.7%), and 4 in 66 (43.4%) patients at six months after the procedure. There were no patients with a satisfaction score of zero.

## Discussion

The mechanism of action of PRF in pain management is currently not clarified with certainty. According to general opinion, radiofrequency has neuromodulatory and anti-inflammatory effects. Microscopic damage is evaluated after exposure to radio waves, as observed in membrane abnormalities and mitochondrial morphology, as well as in interruption and disorganization of microfilaments and microtubules. This ultrastructural pathway occurs more widely in type C and type A nerve fibers. Immune cells are affected by radio waves and inhibit the production of pro-inflammatory cytokines, such as interleukin-1b and interleukin-6 (IL-6). Radiofrequency can induce changes in membranes and intracellular structures, consequently modifying the transmission of action potentials and perception of pain. PRF activates the descending noradrenergic and serotoninergic pain inhibition pathways as well as inhibits excitatory nociceptive C fibers. Microglial activity can be decreased by PRF intervention in DRG. Microglia can cause chronic neuropathic pain through the release of various cytokines and chemokines associated with the transmission of pain signals and also decreased microglial activity may prevent the development of chronic neuropathic pain [11]. On the other hand, it was observed that increased proinflammatory gene expression, such as tumor necrosis factor alpha (TNF-α) and IL-6, was returned to baseline values following PRF therapy [12].

The patients included in the study were with or without history of surgery whose pain was not relieved with more conservative methods. Some of the patients had indications for surgery, but did not want to undergo surgery or had a high risk for surgery due to their medical condition. The VAS scores of the patients who underwent pulsed radiofrequency with multifunctional epidural electrode were significantly lower 10 days and one, three and six months after the procedure as compared to the pre-treatment values.

The prevalence of chronic low back pain by age varies between studies, but it is three to four times more prevalent in the group over 50 years of age than in the group aged 18-30 years [13]. Similarly, in our study, the mean age of 152 patients was 60.8±13.8 years, with the median age being 65 for women and 58 for men. In our study, 63.2% of the patients were female and 36.5% were male. Chronic low back pain is similarly more common among women in literature. A prospective study conducted in patients with persistent lumbosacral radicular pain suggested that young age of individuals and weakness are predictors of decreased success in PRF [14].

In our study, PRF application with multifunctional epidural electrode was most frequently applied to patients with diagnoses of lumbar disc herniation (n=92), spinal stenosis (n=47), and those with spondylolisthesis and spinal stenosis (n=13), respectively. Disc herniation is the most frequent cause of lumbar radicular syndrome in patients under 50 years. After the age of 50, radicular pain often occurs due to degenerative changes in the spine [15].

Pulsed radiofrequency applied to the dorsal root ganglion (PRF-DRG) is an attractive alternative to epidural steroid injection in the treatment of chronic lumbar radicular pain. In pulsed radiofrequency, it is possible to perform target-specific applications and avoid the use of steroids, and thus, eliminate endocrine effects including adrenal suppression, water retention and glucose intolerance. It is known that the injection of steroids, and also of steroid particles, into the artery have potentially serious adverse effects including spinal cord infarction and death [16]. There are very few randomized controlled trials on the use of PRF-DRG for radicular pain. Van Zundert et al. addressed cervical radicular pain in their randomized controlled trial [17]. In their pilot study on lumbar radicular pain, Simopoulos et al. compared patients undergoing conventional RF with patients who had PRF after conventional RF, so it is not an efficacy study [18].

Most of the studies investigating the effects of PRF have been conducted with the transforaminal approach. The multifunctional flexible electrode has advantages over rigid equipment, such as the ability to apply stimulation more closely to the DRG and to infuse drugs into the epidural space [19]. Thanks to the geometric and structural features of the multifunctional electrode, the probe focuses the electric field on the sides instead of the front of the electrode tip, allowing neuromodulation by the reduction of the increase of heat and damage in the tissue [10].

It was observed when the patients were grouped by diagnosis that the VAS scores of the patients diagnosed with LDH were significantly lower 10 days and one, three and six months after the procedure compared to prior to the procedure. There are many studies indicating that the application of PRF to DRG is a safe procedure. Choi et al. reported that 71% of patients with chronic cervical radicular type pain resistant to repeat transforaminal epidural steroid injections (TFESIs) were satisfied with the effectiveness of PRF-DRG [20,21]. 

A prospective randomized trial conducted on patients with radicular pain due to a herniated disc made a comparison between PRF and transforaminal steroid injection, and the VAS scores for cervical and lumbar radicular pain were found to be significantly decreased at the 12th week following the treatments in both groups. There was no statistically significant difference between the PRF and TFESI groups in the VAS, Oswestry Disability Index (ODI) and Neck Disability Index (NDI) scores in any period of the follow-up. Applying PRF to dorsal root ganglion is as effective as TFESI in radicular pain caused by disc herniation, and it allows avoidance from the side effects of the steroids [21]. Based on evidence of very low to low quality, RF denervation is not thought to have an effect different from placebo over the short-term for patients with discogenic low back pain. For the long-term, however, evidence of moderate-quality suggest that RF denervation has lower effect than placebo on pain (numerical rating scale-NRS) and functionality (ODI) [22].

Lumbosacral radicular pain is characterized by a radiating pain in one or more lumbar or sacral dermatomes, and it may or may not be accompanied by symptoms of function loss and other radicular irritation symptoms [15].

A retrospective study investigated patients with radicular syndrome due to disc herniation, spinal stenosis and failed back surgery syndrome (FBSS) that underwent PRF, and reported a significant decrease in analgesic consumption in the groups with disc herniation and spinal stenosis. However, no significant decrease was reported in the analgesic consumption of patients with failed back surgery syndrome [23]. Van Boxem et al. reported a success rate of 29% in 60 patients with chronic lumbar radicular pain. Depending on the success rates reported in the aforementioned studies, it is thought that PRF-DRG may be effective in 30%-50% of patients with chronic lumbar radicular pain [24].

Shanthanna et al. introduced the first randomized controlled study on the effects of PRF in the treatment of chronic radicular pain [25]. The authors demonstrated that PRF had a little effect at four weeks and three months, which was not significantly different from the effect that placebo provided. In that study, the researchers applied PRF with a needle not with an electrode, and the duration of treatment was limited to 120 seconds. These characteristics might have affected the outcomes of the study.

In the prospective randomized trial conducted by Ross et al. on 197 patients, who once underwent lumbar surgery, the epidural scarring caused by lumbar surgery was followed up with magnetic resonance imaging. It was observed in the comparison between epidural scarring and radicular pain that patients who had extensive epidural scarring were 3.2 times more likely to experience recurrent radicular pain [26]. It was observed in our study that the VAS scores were significantly lower on the 10th day and in the first, third and sixth months after the procedure in both groups, regardless of having a history of surgery for pain prior to the procedure. In the study in which Sluijter et al. applied PRF-DRG on radicular pain caused by various aetiologies, 15 of 60 patients were diagnosed with FBSS, and 53% of these patients had a reduction of 2 points in their VAS scores at six months and 40% had a reduction of 2 points in the VAS scores at one year after the procedure [27].

In this study, the pre-procedural analgesic use was 74.3% in all groups, but this rate decreased to 54.6% after the procedure. Cohen et al. showed that the application of PRF to the peripheral nerve is superior to conservative medical treatment in patients with thoracic segmental pain [28].

In studies of PRF application with multifunctional epidural electrode, no major or minor complications have been reported, except for headache lasting for a maximum of one day after treatment [10]. Moreover, no complications have been reported in PRF applications performed on peripheral nerves or in various regions in the spinal area [25]. Similarly, we did not observe any major complications but two patients had headache and dural puncture was seen in one patient.

The greatest problem in the studies investigating PRF is that there is no standardization in PRF parameters, and the inclusion criteria and reports are heterogeneous [29]. In two decades, nearly 200 papers have been published and no complications have been reported. However, there is inadequate evidence in indications; therefore, there is a need for quality randomized controlled studies for identification of optimal parameters for the application of PRF in clinical practice [30].

The major weakness of our study is that it is a retrospective study and does not include a control group. However, it provides valuable information on the efficacy and potential complications of the multifunctional epidural electrode, on which there is limited data in the literature.

In conclusion, it has been demonstrated that pulsed radiofrequency procedure with multifunctional epidural electrode is an effective and safe method in the short- and medium-term for lumbar spinal pain reflected on the low back and legs.

## Conclusions

Low back pain is a condition that affects many people in a certain period of their lives. It has been observed that the prevalence of chronic low back pain has changed over the years due to the change in lifestyles and technology. Primary care for the treatment of low back pain includes non-invasive treatment and non-drug methods, such as patient education and suggestions on active life and exercise. Patients that have low back pain accompanied with severe and progressive loss of motor strength or signs of cauda equine should be immediately referred to surgery. Applying epidural or transforaminal corticosteroid injections and dorsal root ganglion (DRG) radiofrequency have become common next steps in care where conservative treatments are insufficient. In this study, patients, who had low back pain due to various aetiologies, who had no improvement of pain despite medical treatment and other standard treatment methods, were included in the study and it has been demonstrated that pulsed radiofrequency procedure with multifunctional epidural electrode is an effective and safe method in the short- and medium-term for lumbar spinal pain reflected on the low back and legs.
